# CD155 loss enhances tumor suppression via combined host and tumor-intrinsic mechanisms

**DOI:** 10.1172/JCI159825

**Published:** 2022-03-15

**Authors:** Xian-Yang Li, Indrajit Das, Ailin Lepletier, Venkateswar Addala, Tobias Bald, Kimberley Stannard, Deborah Barkauskas, Jing Liu, Amelia Roman Aguilera, Kazuyoshi Takeda, Matthias Braun, Kyohei Nakamura, Sebastien Jacquelin, Steven W. Lane, Michele W.L. Teng, William C. Dougall, Mark J. Smyth

Original citation: *J Clin Invest*. 2018;128(6):2613–2625. https://doi.org/10.1172/JCI98769

Citation for this retraction: *J Clin Invest*. 2022;132(6):e159825. https://doi.org/10.1172/JCI159825

QIMR Berghofer Medical Research Institute recently notified the *JCI* of concerns regarding Figures 4I and 7A and indicated that an independent panel investigation concluded that these figures contain data fabricated by Mark Smyth. In accordance with the institutional recommendation, the *JCI* is retracting this article.

Amelia Roman Aguilera, Matthias Braun, Ailin Lepletier, and Kyohei Nakamura have agreed with the *Journal’s* decision to retract the paper. The remaining authors abstained from commenting or could not be reached.

## Version 1 03/15/2022

Electronic publication

